# First case report of inherited Rubinstein-Taybi syndrome associated with a novel *EP300* variant

**DOI:** 10.1186/s12881-016-0361-8

**Published:** 2016-12-13

**Authors:** María López, Verónica Seidel, Paula Santibáñez, Cristina Cervera-Acedo, Pedro Castro-de Castro, Elena Domínguez-Garrido

**Affiliations:** 1 Molecular Diagnostics Laboratory, Fundación Rioja Salud, Logroño, La Rioja Spain; 2 Clinical Genetics, Department of Pediatrics, Hospital General Universitario Gregorio Marañón, Madrid, Spain; 3 Section of Neuropaediatrics, Hospital General Universitario Gregorio Marañón, Madrid, Spain; 4 Center for Biomedical Research (CIBIR), C/Piqueras 98, C.P. 26006 Logroño, Spain

**Keywords:** Rubinstein-Taybi syndrome, RSTS, *EP300*, Inheritance, Familial-RSTS, Case report

## Abstract

**Background:**

Rubinstein-Taybi syndrome (RSTS; OMIM #180849, #613684) is a rare autosomal dominant genetic condition characterized by broad thumbs and halluces, facial dysmorphism, short stature and variable degree of intellectual disability. RSTS is associated with mutations in *CREBBP* and *EP300* genes in 50–60% and 5–8% of cases, respectively. The majority of cases are *de novo* heterozygous mutations.

**Case presentation:**

Here we describe a familial RSTS case, associated with a novel *EP300* mutation. The proband was a 9 years old female, with mild learning difficulties. Her mother, who also had learning difficulties, was found to have short and broad thumbs. MLPA and panel-based NGS of *CREBBP* and *EP300* were performed. A novel heterozygous frameshift mutation in exon 31 of the *EP300* gene (c.7222_7223del; p.(Gln2408Glufs*39)) was found in both.

**Conclusions:**

This case represents the first case of inherited *EP300*-RSTS. The location of the frameshift deletion not affecting HAT domain and PHD finger, could explain the mild phenotype and the well-preserved intelligence. These patients are mildly affected, and this case highlights the possible missed diagnosis. We would recommend molecular testing of apparently healthy parents, and in the case of inherited mutations, of all adult first degree relatives at risk.

**Electronic supplementary material:**

The online version of this article (doi:10.1186/s12881-016-0361-8) contains supplementary material, which is available to authorized users.

## Background

Rubinstein-Taybi syndrome (RSTS; OMIM #180849, #613684) is a rare (1:125000) complex neurodevelopmental disorder characterized by broad thumbs and halluces, facial abnormalities (downslanted palpebral fissures, low hanging columella, high palate, grimacing smile, and talon cusps), postnatal growth delay and intellectual disability (ID) [2, 10]. In addition, RSTS patients have a slightly increased predisposition to cancer [9]. RSTS is an autosomal dominant condition and the vast majority of cases occur sporadically due to *de novo* heterozygous mutations, being vertical transmission extremely rare [7]. So far, Bartsch et al. [3] described only five familial cases of RSTS, consistent with autosomal dominant inheritance, and recently another possible case has been described in India [15].

Two genes have been implicated with RSTS: *CREBBP*, located on chromosome 16p13.3, encoding a CREB-binding protein (CBP), and *EP300,* which maps to 22q13.2 and encodes E1A-associated protein p300. They are very similar and function as transcriptional coactivators in the regulation of gene expression mediating many of the same signalling pathways. CBP and p300 are highly conserved and have potent histone acetyltransferase (HAT) activity. Consequently, there is a direct link between loss of acetyltransferase activity and RSTS suggesting an aberrant chromatin regulation [17, 18]. Despite their high sequence similarity (>70%), CBP and p300 have differences in their respective functions [1]. RSTS is caused in approximately 50–60% of cases by mutations of the *CREBBP* gene (RSTS 1, OMIM #180849), and by *EP300* gene mutations (RSTS 2, OMIM #613684) in 5–8%. To date, 246 disease causing mutations in the *CREBBP* gene have been reported to cause RSTS [7]. In contrast, only 23 RSTS patients with *EP300* mutations, and a total of 34 mutations, have been described in this gene [10, 14]. The mutations described in both range from point mutations to whole gene deletions and chromosome rearrangements, and spread across the entire length of the genes.

Several reasons are being postulated for the lower detection rate of *EP300* mutations: a lower mutation rate; underdiagnosis at similar mutation rates following the generally milder phenotype in cases of RSTS with *EP300* mutation; or misdiagnosis in cases with severe phenotype resembling other syndromes with congenital malformations such as Cornelia de Lange syndrome (CdLS) which interestingly is caused by alterations in other chromatin associated proteins [1, 9, 13, 17].

In this case report we describe an inherited RSTS case associated with a novel *EP300* mutation, representing the first familial case found in Spain.

## Case presentation

**Fig. 1:**
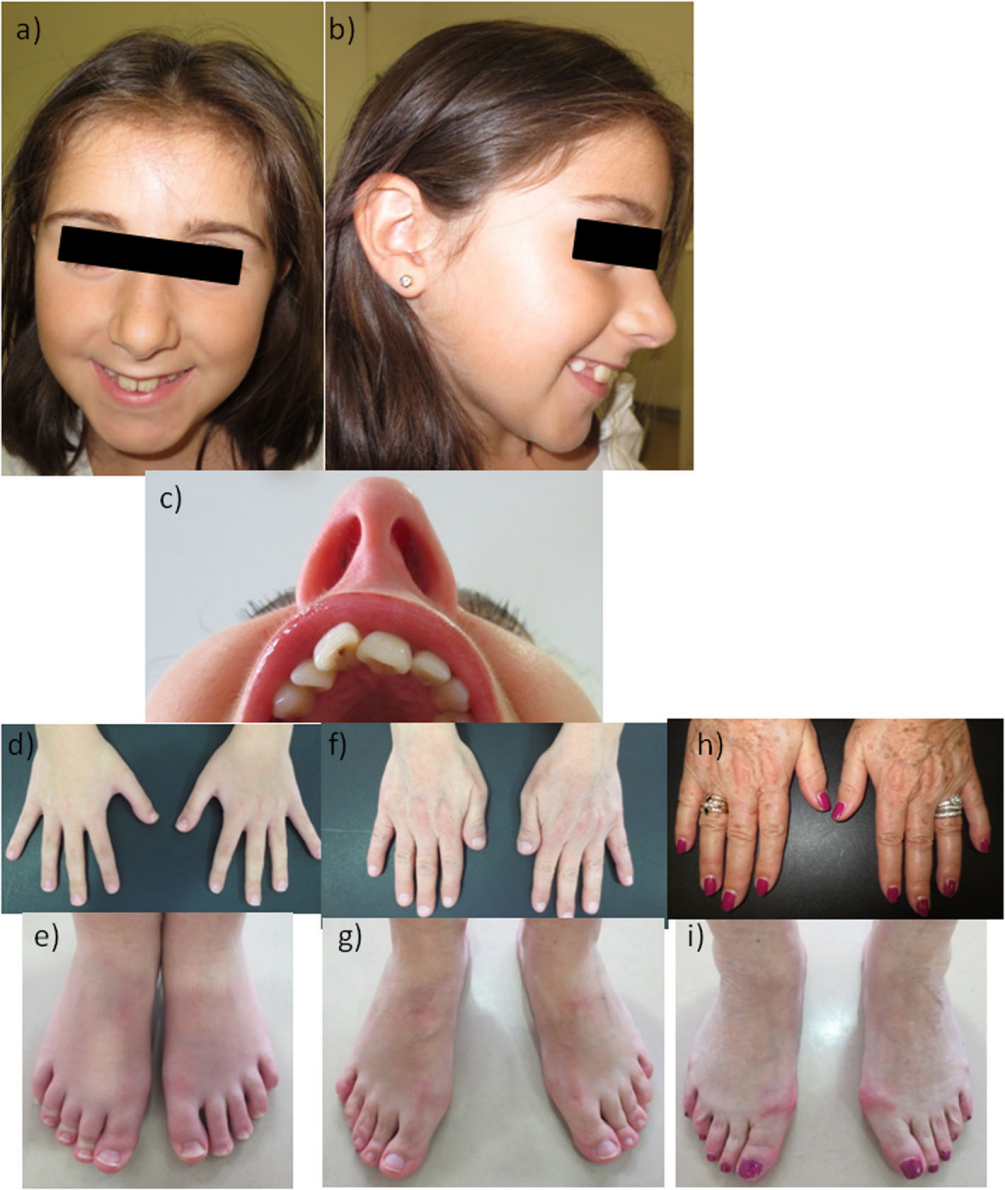
Images showing classical RSTS features of patient including thickened and low hanging columella (**a**, **b**), and detail of mother’s talon cusp at an upper incisor (**c**). Photographs showing normal thumbs and halluces of the proband (**d**, **e**), short and broad but not angulated thumbs of her mother (**f**, **g**) and her grandmother (**h**, **i**)

Proband was a 9-year-old girl, referred to the Genetics Clinic by the neuropediatrician who had been following her for mild learning difficulties. RSTS was suspected after seeing her mother, who also had learning difficulties and had short broad thumbs. Our patient was born at term via Cesarean section (pelvi-fetal disproportion) after an uneventful pregnancy. Both, weight and length at birth were low: 2.3 kg (−2.38 SD) and 46 cm (−2.07 SD), respectively. Occipitofrontal head circumference (OHC) at birth was not recorded. The girl’s development was within normal range in the first years of life but increasing learning difficulties were noted at primary school. She is at her expected grade with educational support. On physical examination the proband’s weight was 21.4 kg (−1.74 SD), her length 119 cm (−3.15 SD on Spain 2000 growth chart), and her OHC 48.7 cm (−2.9 SD). She had a thickened and low hanging columella. Her hands and feet (thumbs and halluces) were quite normal (Fig. 1. Phenotype table (Additional file 1)).

The mother (42 y.o.) had short stature (141 cm (−3.87 SD)) and proportionately small OHC (49 cm (−4 SD)). She had struggled at school but managed to get the Secondary School Certificate. Nowadays she is working as a check-out in a supermarket. Her general health is good. She is a single mother of two children: the proband and a healthy 7 year old boy. She had slightly posteriorly rotated ears, a prominent nose with high nasal bridge, low hanging columella, and talon cusp at an upper incisor. Her thumbs were short and broad but not angulated and her halluces were normal (Fig. 1. Phenotype table (Additional file 1)). Proband’s maternal aunt and grandmother also had short stature and proportionate OHC but normal intelligence and normal hands and feet. The grandmother’s length was 142.1 cm (−3.5 SD) and her OHC 52.5 cm (−1.9 SD). Maternal grandfather died aged 50 from liver disease.

Previous assessments of the proband included a hand X-ray for bone age, which was according to chronological age, and endocrine tests with normal levels of growth hormone in two stimulation tests. A brain MRI scan showed a pineal cyst (9 × 9 × 5 mm) and no other anomalies.

Clinical data, samples and photographs were obtained after written informed consent. This work has been approved by the Committee for Ethics in Clinical Research in La Rioja (CEICLAR).

Blood samples from the proband, her mother and her grandmother were collected in EDTA tubes. DNA was extracted using QIAamp DNA Mini Kit (QIAGEN) following the manufacture’s protocol. MLPA of *CREBBP* and *EP300* was performed (P313 and P333 Kit, MRC-Holland). Panel-based next generation sequencing (NGS) of *CREBBP* and *EP300* genes was carried out. Briefly, libraries encompassing exons and introns of *CREBBP* and *EP300* genes were prepared using the SureSelect^XT2^ Custom kit (Agilent) and sequenced to generate 150 bp single reads. The resulting reads were mapped to the human genome hg19 using BWA (version 0.7.12). Sequence variants were called using the Genome Analysis Toolkit (GATK) version 3.3 and called variants were annotated with Annovar. ExAC browser of Broad Institute, 1000 Genomes database and dbSNP138, as well as, the Human Gene Mutation Database (HGMD), Leiden Open Variation Database (LOVD) and ClinVar databases were checked to assess the presence/absence of detected alterations in variations repositories.

## Results and discussion

MLPA did not reveal any deletion within the *CREBBP* or *EP300* genes. Only one variant was identified in the panel-based NGS of *CREBBP* and *EP300* genes: a heterozygous mutation in exon 31 of the *EP300* gene (RefSeq NM_001429.3: c.7222_7223del; p.(Gln2408Glufs*39)). As in the proband, this variant was also found in the heterozygous state in the mother as is shown by the number of reads (238 reads with deletion of a total of 486, for the proband; and 219/485 for her mother). This finding was confirmed by Sanger sequencing. This variant was not found in 100 healthy controls and it is not present in 1000G, ExAC and dbSNP, indicating that this variant is not common in population. To the best of our knowledge this variant has not been previously described, and it is not included in ClinVar, HGMD or LOVD. The variant has now been included in ClinVar database (SCV000266471) and in LOVD (individual #00064625). The deletion generates a frameshift that leads to loss of the original stop codon and results in a prolonged protein 31 aminoacids longer. This variation is considered to be pathogenic/likely pathogenic, according to ACMG interpretation: null variant in a gene where LOF is a known mechanism of disease, absent in population databases, protein length changing variant, and patient’s phenotype highly specific for gene [12].

According to the literature, point mutations are widespread along the whole *EP300* gene without any remarkable hot spot, being frameshift mutations the most common found, as is our case [10, 14]. Likewise, genotype-phenotype correlations indicated that patients with larger deletions did not always have a more severe phenotype than those with smaller deletions or point mutations. On the other hand, it seems that mutations that do not alter HAT domain could explain non-classical RSTS cases [3, 18]. In this sense, the location of the frameshift deletion described in this report, very close to the 5′-end of *EP300* gene, not affecting HAT domain and PHD finger, could explain the mild phenotype and the well-preserved intelligence.

Addressing the lower frequency of *EP300* mutations (*EP300* mutations are 10 times less frequent than *CREBBP* mutations), it may be possible that *EP300* gene had a lower mutation rate than *CREBBP*, nevertheless, many more polymorphisms have been found in this gene, including some that lead to amino acid changes [13]. Hence, *EP300* mutations might account for a higher frequency of RSTS patients than previously thought. However, because of phenotypic variability such patients are not ascertained and not studied.

Molecular analysis was performed in mother and grandmother and it was positive in the mother and negative in the grandmother, confirming the inherited character of this mutation, suggesting three possible options: 1) the mutation appeared *de novo* in the mother and was passed on to her daughter, 2) the grandmother could have germline mosaicism, that could not be corroborated or 3) the grandfather, who presented broad thumbs, could also be a carrier of the mutation. Thereby, despite the inherited confirmation of this RSTS case, it has not been possible to determine the exact point where the mutation appeared.

Familial RSTS is extremely rare, in this regards, between 1000 and 2000 cases of RSTS have been reported but only 11 cases of familial RSTS have been described [3–6, 8, 11], and are most likely linked to somatic mosaicism. Moreover, in all the cases in which the molecular cause was determined, it was associated with the *CREBBP* gene. Recently, Tamhankar et al., reported a patient with mutation in *EP300* gene inherited from a healthy mother [15]. Also Negri et al., and Wincent et al., [10, 16] found a missense variation in both, a patient and his healthy mother and father, respectively, but both were considered as not pathogenic. Although Tamhankar et al. suggested the possibility of low penetrance, these variants should be considered not pathogenic, since they have been detected in healthy parents. Moreover, only one *EP300* missense mutation has been previously observed in RSTS patients [10], indicating that this type of change is not probable as causative of RSTS.

Affected children tend to have more pronounced features than their affected parent in familial RSTS [3]. Conversely, in our case it was the mother who showed the characteristic thumbs, facial features and dental anomalies, giving rise to the suspicion of RSTS diagnosis. On the other hand, the familial short stature with proportionately small HC turned out not to segregate with the *EP300* mutation and may therefore be associated with other genetic factors not studied.

This study broadens the number of phenotypic *EP300*-patients described to 24 providing additional knowledge about the phenotype of these RSTS-2 cases. In general, this cohort presents mild presentation of the phenotype, and also few phenotypic peculiarities, what is attested by the age at diagnosis, usually higher in *EP300* patients [9, 10, 14]. Our observations are in accordance with this, since the proband was diagnosed at the age of 9, and her mother was not diagnosed, until this study.

According to the literature, these patients usually present a mild presentation of the syndrome. It is frequently reported in these RSTS-2 patients classic facial RSTS features (prominent nose with low-hanging nasal septum, downward slanting palpebral fissures, etc.); variable degree of intellectual disability, ranging from mild to moderate (no patient has a severe impairment); mild skeletal phenotype, even if there are cases described with normal thumbs and toes, it is common to find broad thumbs and big toes although with no radial deviation of them [1, 10, 16]. In our case, mother and proband showed mild learning difficulties, but the mother was able to lead a normal life. Both showed facial characteristics of RSTS although they were more evident in the mother. Also, the mother had broad but not angulated thumbs, whereas the child had normal thumbs and halluces.

## Conclusion

In summary, this study presents the first case of inherited *EP300*-RSTS. This report underscores the possible lack of diagnosis in these patients with non-classic presentations, as is the case of patient’s mother. Therefore, variable clinical expression exists in RSTS and normal functioning individuals may carry disease-associated mutations, even in a non-mosaic state. These findings have implications for genetic counselling and we would recommend molecular testing of apparently healthy parents, and in the case of inherited mutations, of all adult first degree relatives at risk. Thus, it is important to highlight that since most individuals with RSTS due to *EP300* mutation are mildly affected, this entity is likely underdiagnosed and special attention should be paid.

## Additional file

Additional file 1: Phenotype table. Phenotype found in patient and her mother. (XLS 26 kb)

## Abbreviations

ACMG: American College of Medical Genetics and Genomics
CBP: CREB-binding protein
CdLS: Cornelia de Lange syndrome
CEICLAR: Committee for ethics in clinical research in La Rioja
GATK: Genome analysis toolkit
HAT: Histone acetyltransferase
HGMD: Human gene mutation database
ID: Intellectual disability
LOVD: Leiden open variation database
MRI: Magnetic resonance imaging
NGS: Next generation sequencing
OHC: Occipitofrontal head circumference
PHD: Plant homeodomain
RSTS: Rubinstein-Taybi syndrome
